# Models for Implant-Induced Capsular Contracture Post Breast Cancer Surgery

**DOI:** 10.1007/s11538-023-01236-2

**Published:** 2023-12-13

**Authors:** Cheryl Dyck, Kathryn V. Isaac, Leah Edelstein-Keshet

**Affiliations:** 1 Insight Math Unincorporated, Port Moody, BC, Canada; 2 Department of Surgery, Faculty of Medicine, University of British Columbia, Vancouver, BC Canada; 3 Department of Mathematics, University of British Columbia, Vancouver, BC V6T 1Z2 Canada

**Keywords:** Reconstructive breast surgery, Cellular response to implant, Inflammation, Mathematical model, Bistability

## Abstract

Capsular contracture is a painful deformation of scar-tissue that may form around an implant in post-breast cancer reconstruction or cosmetic surgery. Inflammation due to surgical trauma or contamination in the tissue around the implant could account for recruitment of immune cells, and transdifferentiation of resident fibroblasts into cells that deposit abnormally thick collagen. Here we examine this hypothesis using a mathematical model for interacting macrophages, fibroblasts, myofibroblasts, and collagen. Our model demonstrates that cellular response can, together with inflammatory cell recruitment, account for prognoses.

## Introduction

Capsular contracture (CC) is one of the most common complications following breast implant surgeries (Headon et al. 2015). In one retrospective study, the overall rate of occurrence was 11.8%, with a 62.5% rate for breast cancer patients undergoing radiotherapy (Loreti et al. 2020).

In the clinical practice of one of the authors (KVI), breast reconstruction is offered to patients with breast cancer to improve their quality of life as survivors. When a patient experiences a capsular contracture as a complication, the benefits derived from the reconstruction are limited by the need for subsequent re-operations and possible failure of the reconstruction. Although there have been advances in biomaterials used in plastic surgery and breast reconstruction to prevent CC, we have not advanced our understanding its etiology. This has limited our ability to prevent and appropriately treat CC when it occurs. Infectious and aberrant inflammatory processes are thought to cause CC, resulting in many complex cellular and chemical interactions that need to be accounted for when conceptualizing and testing hypotheses for the development of CC.

This project arose from a call by a practicing surgeon (KVI) to seek a different strategy for tackling the problem of understanding CC etiopathogenesis. Given that there has been decades of bench and clinical research on individual components associated with CC, it is reasonable to believe that evidence could be compiled, and could lead to mathematical modeling that addresses this complex biologic process to provide insights. This paper represents the first step in such a program.

After reconstructive breast surgery, an inflammatory response to the implant, called a Foreign Body Reaction (FBR), initiates immunogenic formation of a capsule of collagenous extracellular matrix (ECM) to exclude the foreign body. While pliant capsule formation is a normal self-limited response, in capsular contracture, this process becomes pathological. The severity of the pathology is quantified with a scoring system denoted “Baker Grade” (BG); BG 1–2 are mild, but BG 3–4 describe capsules that have become firm, contracted, visibly deformed, and painful. The pathology develops over a span of months to years, is generally unilateral, and occurs more frequently in the setting of implant-based reconstruction for breast cancer as compared to cosmetic breast augmentation. Radiation therapy increases the likelihood of capsular contracture (Ho et al. 2014). Although the cause is uncertain, theories postulate that radiation (1) impairs the local immunologic response of the tissue, and increases risks of infection that can result in CC (Araco et al. 2009; 2) causes fibrosis of the dermis (Hardy Abeloos et al. 2023) or the pectoral muscle (Sobti et al. 2020) that induces fibrosis nearby.

Treatment for CC generally requires surgical excision of the capsule and replacement of the implant. Recurrence varies between 0–50% depending on treatment, whether by partial or total removal of the contracted capsule, with or without a change in the placement of the new implant. The lowest recurrence rates occur if the entire capsule is removed and the implant is placed in a new anatomic plane relative to the original placement (Boyd et al. 2023). In a retrospective study, Montemurro et al. (2021) found a 7.5% recurrence of CC two years after the capsule was surgically removed, and a new implant inserted in a new position. Since aberrant collagen deposition is a hallmark of CC, possible treatment by collagenases has been considered. Although this therapeutic intervention was previously clinically indicated for another fibrotic disease, Dupuytren’s, it remains under investigation for the treatment of CC (Fischer et al. 2015; Diehm et al. 2019).

The cellular, biochemical, surgical, and biomaterial factors that cause normal capsule formation to proceed to the pathological case have been studied extensively, with many candidate theories proposed. However, identifying what differentiates patients that develop CC compared to those that do not remains elusive. Elucidating specific triggers or conditions, whether from external factors after surgery or from a patient’s genetic, physiolological, or biochemical profile, would help to predict a specific patient’s risk profile, or better yet, develop targeted therapy to prevent CC. Here our goal is to investigate the early stages of CC development by exploring the mechanisms of cellular recruitment and collagen production, with an aim of gaining insights into possible preventative measures.

Previous investigators have proposed models related to collagen turnover  (Sáez et al. 2013), fibroblast and collagen orientation (Dallon and Sherratt 1998), collagen lattice contraction (Dallon et al. 2014), foreign body reaction and collagen growth (Su et al. 2011), general wound healing (Cumming et al. 2010; Flegg et al. 2015), and the macrophage-fibroblast relationship (Jansen et al. 2019). Structural and mechanical aspects of implants (Borau et al. 2011) and other collagen matrices have also been studied. We mention the works on buckling of capsules (Ben Amar et al. 2015), morphoelastic models (Egberts et al. 2021), stress–strain relationships within the ECM (Ramtani 2004), contracture of burn scars (Koppenol et al. 2017), and force-balance approach (Olsen et al. 1995). Many of these, and other models for wound healing have been described in the authoritative review by Menon and Flegg (2021).

## Biological Background

**Fig. 1:**
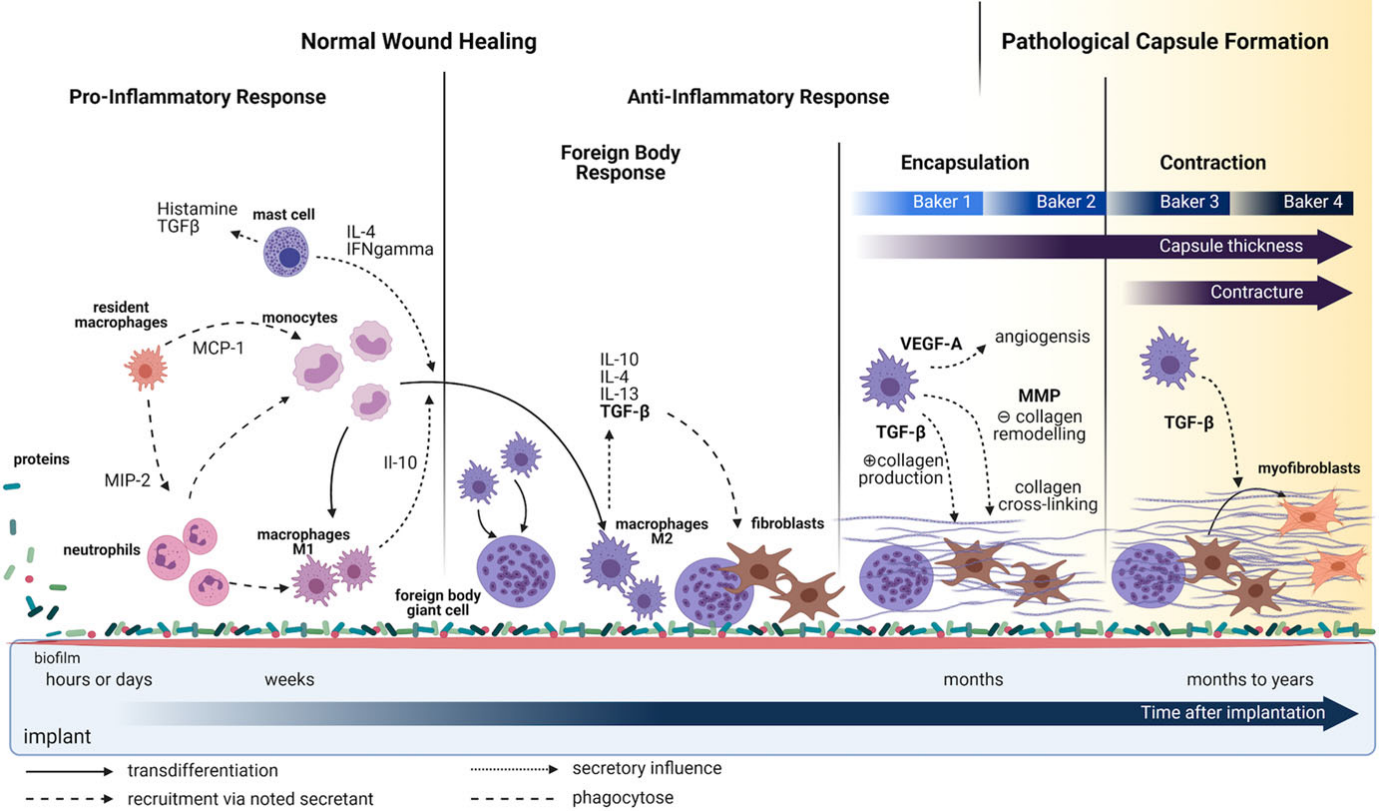
Time sequence (left to right) of response to implant surgery. An immune response causes a provisional matrix to be adsorbed to the implant surface and tissue-resident immune cells recruit neutrophils. Activated pro-inflammatory macrophages are involved in an immune cascade, ultimately recruiting fibroblasts that modify the collagen matrix. Fibroblasts transdifferentiate into myofibroblasts that produce contractile forces. Collagen remodeling sets the contracted ECM. Scheme made with Biorender

The cellular and chemical processes associated with the response to an implant are shown schematically in Fig. 1. The process is complex, involving a cascade of cell recruitment, chemical signaling, collagen deposition, and tissue contraction. In this paper, we model one specific aspect of the foreign-body and early encapsulation responses, namely the recruitment of cells and collagen deposition. A caricature of the process is shown in Fig. 1, but we simplify, and focus on a subset of implicated cell types.

We consider the interactions of the fibroblasts, myofibroblasts, and macrophages. Fibroblasts and myofibroblasts are both collagen producing cells. Myofibroblasts also secrete more collagen than fibroblasts (Santiago et al. 2010). Both cell types are contractile, developing intracellular actin-myosin stress fibers (Li and Wang 2011; Klingberg et al. 2013). Myofibroblasts express $\alpha$-Smooth Muscle Actin ($\alpha$-SMA) (Noskovicova et al. 2021) which allows them to undergo greater contraction than fibroblasts.

Macrophages, cells of the innate immune system that respond to infection and foreign bodies, secrete cytokines such as TGF-$\beta$ that recruit other cells (Pakshir and Hinz 2018) including fibroblasts. Macrophages promote the transdifferentiation of fibroblasts into myofibroblasts.

Collagen, a major component of the extracellular matrix, is the main constituent of capsules formed around breast implants. Higher concentration and alignment of collagen increases the stiffness of the capsule, and is associated with more severe capsular contracture (McDougall et al. 2006). Collagen stiffness can promote the transdifferentiation from fibroblasts to myofibroblasts (D’Urso and Kurniawan 2020; Liu et al. 2010).

Numerous inter-related cellular and mechanical positive and negative feedbacks are involved in the development of the peri-implant capsule. Here we investigate the feedbacks to cell recruitment and transdifferentiation. The mechanosensitivity and contractility of cells will be addressed in a separate work.

We focus on the subset of cells that depict the strength of the immunological response, its direct downstream effect on the tissue cell composition, and feedbacks that control the level of secreted collagen. We derive a mathematical model of three cell types, and simplify it to a minimal model that has the same inherent behaviour. By stripping away some of the complexity, we focus on the key feedbacks that determine the normal versus pathological response to the implant, showing conditions that promote capsular contracture.

## Full Cell-Collagen Mathematical Model

We consider a time-dependent model for cells and collagen in the vicinity of an implant. At this stage, the spatial distribution of cells is not explicitly considered, but the recruitment and egress (or turnover) of cells is modeled.

**Fig. 2:**
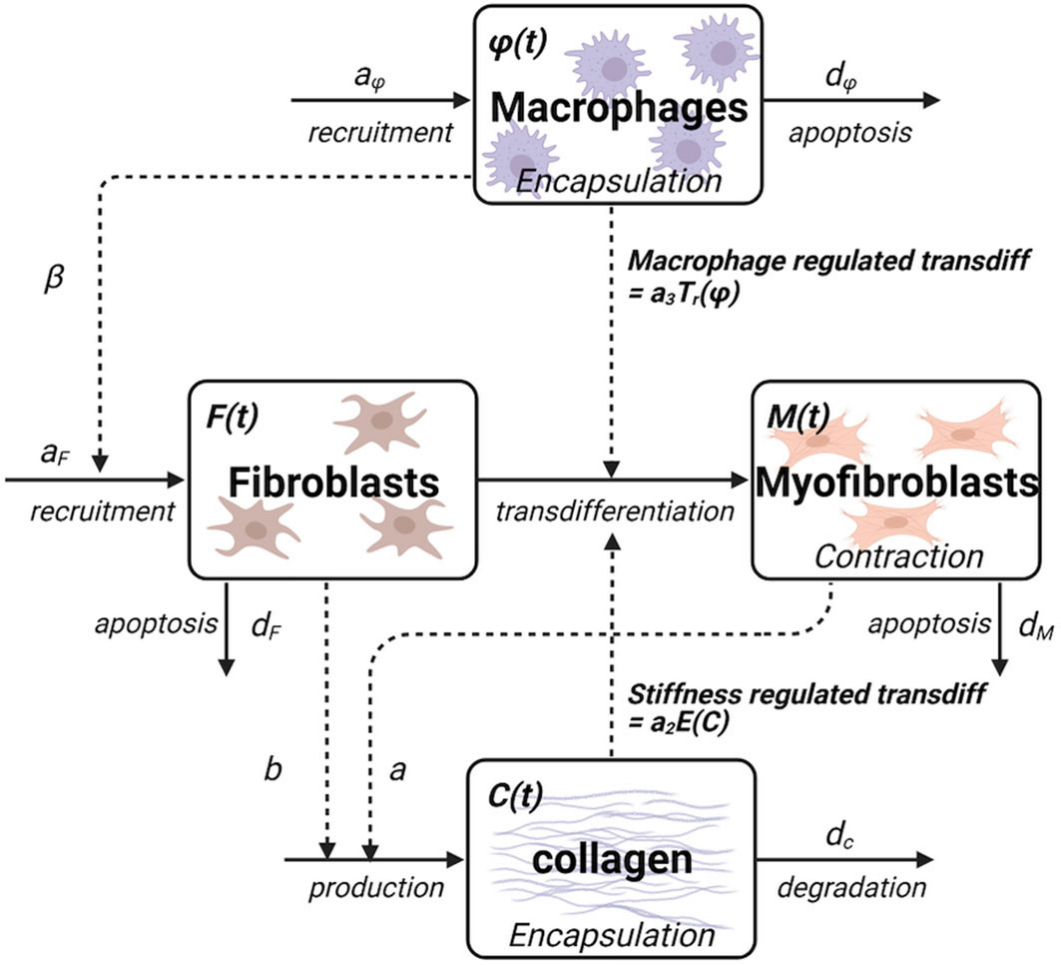
Schematic diagram for the model. The physiology of capsule formation is reduced to several key elements in this schematic diagram. The implant surgery initiates an immune response and macrophage recruitment which leads to recruitment of fibroblasts and myofibroblasts that produce collagen. The amount of collagen affects capsule stiffness and amplifies the transdifferentiation of fibroblasts to myofibroblasts. Scheme made with Biorender

In the following models, we denote by $\phi (t), F(t)$, and *M*(*t*) the macrophage, fibroblast and myofibroblast densities, respectively; *C*(*t*) represents the density of collagen. We describe the interactions of cells in the schematic diagram shown in Fig. 2.

To construct the model, we make the following basic assumptions.

1. Macrophages and fibroblasts are recruited to the implant site (rates $a_\phi , a_F$). These cells also have some turnover rate ($d_\phi , d_F$) that could be due to death, egress, or transdifferentiation (to other cell types not modeled). We take the turnover rates to be constant.
2. The macrophage recruitment rate $a_\phi$ is associated with the intensity of the initial immune response, and controls the influx of cells that leads to the cascade shown in Fig. 2. Hence, this parameter will be an important tunable property of the system.
3. Recruitment of cells could be affected by feedback between cells, including chemical or other stimuli. Specific assumptions are made in what follows.
4. Myofibroblasts are formed by a process of transdifferentiation of fibroblasts (rate $a_T$), for which feedback from macrophages and/or from collagen stiffness could play a role. (We explore specific feedbacks in our analysis.)
5. Collagen is produced by both fibroblasts and myofibroblasts (rates *a*, *b*), with the latter making a greater contribution. Low background level collagen degradation by other cells is depicted by the turnover parameter $d_C$.

With these assumptions, the essential model structure is given by

1a $$\begin{aligned} \frac{d\phi }{dt}&= a_\phi - d_\phi \phi , \end{aligned}$$

1b $$\begin{aligned} \frac{d F}{dt}&= a_f - a_T F-d_F F,\end{aligned}$$

1c $$\begin{aligned} \frac{d M}{dt}&= a_M+ a_T F-d_M M,\end{aligned}$$

1d $$\begin{aligned} \frac{d C}{dt}&= aM+bF - d_C C. \end{aligned}$$

The default model structure is based on the idea that all components should have (at least one) steady state solution. Each model equation is of the general form $dy/dt = a - d y$. For constants $a,d>0$, this implies a steady state solution ($dy/dt=0$) when $y=a/d>0$, avoiding explosive exponential growth. Hence, the presence of pathology would be associated with the level (and multiplicity) of steady states, not with blowup or explosive growth.

Our hypothesis for the pathology of capsular contracture is that *under suitable conditions, an elevated immune response (increase in* $a_\phi$), *could account for the CC pathology in some cases.* To explore this hypothesis, and predict conditions for CC, we make further assumptions about the model.

### Simplest Linear Case is Unrealistic

First, suppose that all parameters $a_i, d_i$ in the model are constants. Then, in this case, the system (1) is simply linear. Hence, for any parameter setting, there would be a single stable steady state, where the densities of cells and collagen are simply dependent on ratios of recruitment and turnover. (For example, $\phi _{SS}=a_\phi /d_\phi$, etc.) This also means that in the linear model, changing parameters just shifts the single steady state along some continuous gradation, but does not account for (a) distinct endpoints of normal vs pathological outcomes and (b) the fact that an immunological insult can lead to CC in women who were not previously observed to develop the condition. This appears contrary to clinical data, where CC presents as a severe disfiguring outcome, having progressed from a normal pliable capsule to contracted firm capsule. Further, the effect of an immunological insult will emerge as critical in the revised bistable version of the model.

### Feedback and the Nonlinear Model

Considering that a linear model fails to account for a discrete dichotomy between normal and pathological states, we add several assumptions to create the next version of the model.

1. Fibroblast recruitment is augmented by macrophage density (Sapudom et al. 2021). (This could stem from cytokines or cell-cell interactions.) We take a simple form for this dependence, assuming that
  $$\begin{aligned} a_f=a_F+\beta \phi . \end{aligned}$$
  where $a_F$ is the basal rate of recruitment, and $\beta$ the enhanced macrophage-induced rate of fibroblast recruitment per macrophage.
2. Fibroblast transdifferentiation rate is influenced by macrophage density (Noskovicova et al. 2021), as well as by collagen stiffness (Tai et al. 2021), that is,
  $$\begin{aligned} a_T=a_2 E(C) +a_3 T_r(\phi ), \end{aligned}$$
  with the parameters $$ a_2, a_3$$ governing the relative contributions of the two feedbacks. [$$a_2=0$$ means that only macrophages can affect the process, whereas $$a_3=0$$ means that only collagen can do so.]
3. The influences of macrophages and collagen, respectively, on transdifferentiation, denoted $T_r(\phi )$ and *E*(*C*), are each saturating functions with a nonlinear switch-like characteristic.

With the above assumptions, the model becomes

2a $$\begin{aligned} \frac{d\phi }{dt}&= a_\phi - d_\phi \phi , \end{aligned}$$

2b $$\begin{aligned} \frac{d F}{dt}&= a_F+\beta \phi - (a_3 T_r(\phi ) +a_2 E(C))F-d_F F, \end{aligned}$$

2c $$\begin{aligned} \frac{d M}{dt}&= a_M+ (a_3 T_r(\phi ) +a_2 E(C)) F-d_M M, \end{aligned}$$

2d $$\begin{aligned} \frac{d C}{dt}&= aM+bF - d_C C. \end{aligned}$$

where we take the macrophage and collagen influence on transdifferentiation to be

2e $$\begin{aligned} T_r(\phi )=\frac{\phi ^{m_n}}{\phi _0^{m_n}+\phi ^{m_n}}, \quad E(C)= \frac{C^n}{C_0^n+C^n}. \end{aligned}$$

In this first model, we do not consider the structure or mechanical properties of the collagen within the capsule directly. Rather, we assume that the collagen density is correlated with capsule stiffness and risk of developing CC. $T_r(\phi )$ and *E*(*C*) are Hill functions that increase monotonically and saturate to the value 1 (as $\phi$, respectively *C* grow large). The parameters $\phi _0, C_0 >0$ are, respectively the macrophage and collagen densities that result in a 50% response (i.e., $T_r(\phi _0)=0.5 = E(C_0)$. The Hill coefficients $m_n,n$ control the sharpness of the response; larger values of these parameters result in a sharper switch-like response (“off” when $\phi <\phi _0$, “on” when $\phi >\phi _0$.)

### Reduced Toy Model

Detailed values of parameters that appear in Model (2) are not readily ascertained for the response to breast implants. Hence, before studying the full model, we consider a reduced version whose qualitative behaviour can be more easily understood.

To do so, we combine some variables and use timescale arguments to simplify the set of equations. We make the following assumptions.

- Fibroblasts and myofibroblasts are both collagen-producing cells (with different relative contributions). We consider an average collagen production rate and lump *F* and *M* into a single effective population, again denoted *M*.
- Macrophage infiltration of tissue post-surgery is likely to be relatively rapid compared to timescale of other responses. Hence, for the toy model, we assume that the density of macrophages settle into a quasi steady state value ($\phi _\text {QSS} \approx a_\phi /d_\phi$) on the timescale of the formation of the capsule. Then, we treat the recruitment rate of cells *M* as a (tuneable) constant, amplified by a collagen-dependent rate.

**Fig. 3:**
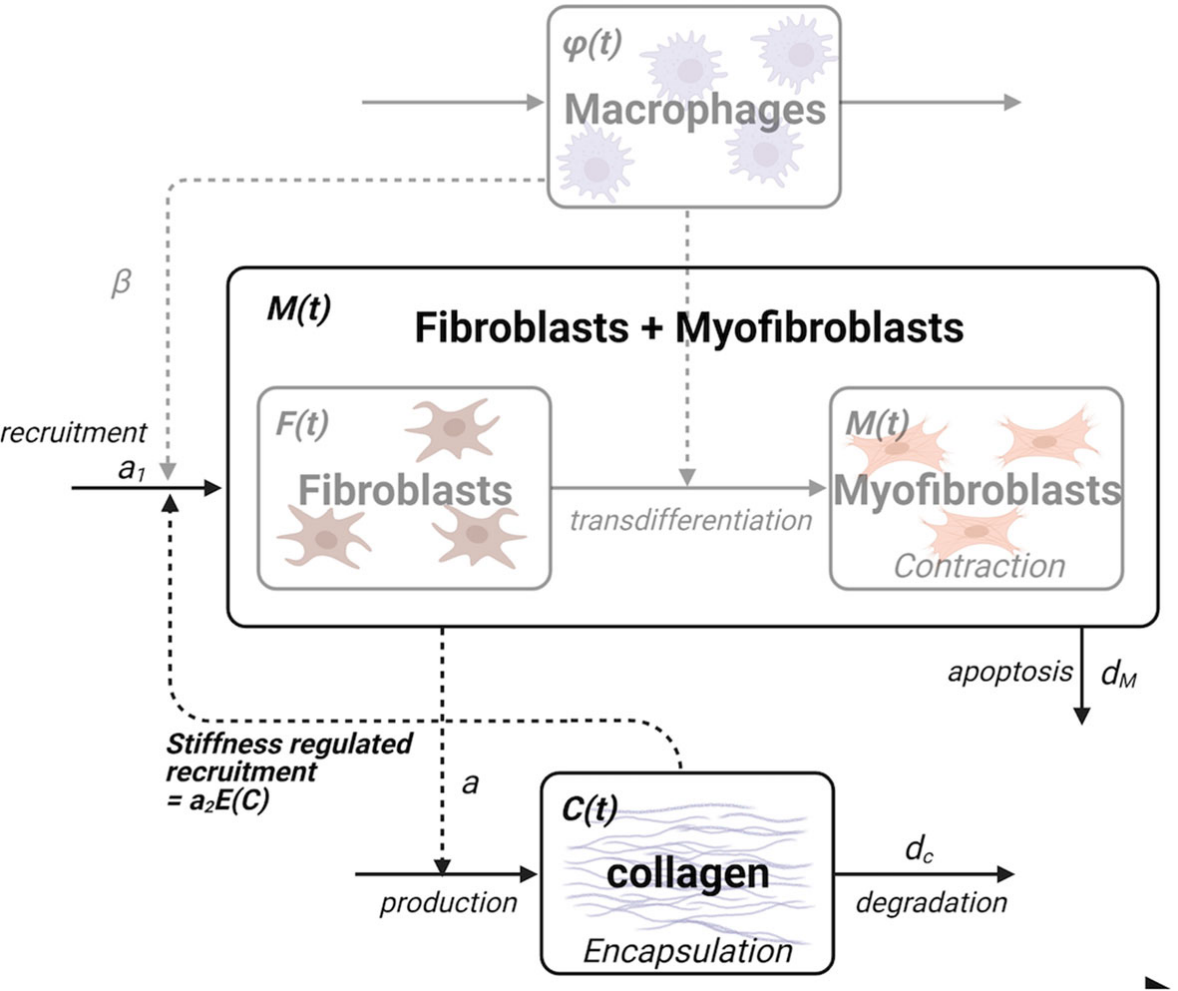
Schematic for reduced model. The fibroblasts and myofibroblasts are lumped into a single collagen-secreting population. The recruitment rate of fibroblasts, $a_1$ is now the tuning parameter (previously dependent on macrophage density). Scheme made with Biorender

The above simplifications are shown in Fig. 3. The resulting model equations take the form

3a $$\begin{aligned} \frac{d M}{dt}&= a_1+ a_2E(C) -d_M M, \end{aligned}$$

3b $$\begin{aligned} \frac{d C}{dt}&= aM - d_C C, \end{aligned}$$

where

3c $$\begin{aligned} E(C) = \frac{C^n}{C_0^{n}+C^{n}} . \end{aligned}$$

As before, *E*(*C*) is the amplified cell recruitment in response to collagen levels. This nonlinear feedback is key to the normal versus pathological response.

## Simulations and Results

### The Toy Model Provides Insight

The model (3) now has only a few parameters, and their relative values can be more directly understood. As there are only two key variables in model (3), we can use the *MC*-phase plane to understand its qualitative behaviour.

First, we selected a timescale associated with cell turnover time, which is equivalent to setting $d_M\approx 1$. We consider a scenario wherein collagen remodeling and turnover is on a similar timescale $d_C\approx 1$. We pick a relatively small basal cell recruitment in the absence of feedback (small $a_1$) and a potentially large feedback effect (large $a_2$) to demonstrate ideas. The results then depend on the sensitivity of the response, which is set by the collagen Hill parameter $C_0$ and the Hill coefficient *n*.

Nullclines of the model are the two curves

$$\begin{aligned} M= \frac{1}{d_M} \left( a_1+ a_2 \frac{C^n}{C_0^{n}+C^{n}}\right) ,\quad C= \frac{a}{d_C}M. \end{aligned}$$

**Fig. 4:**
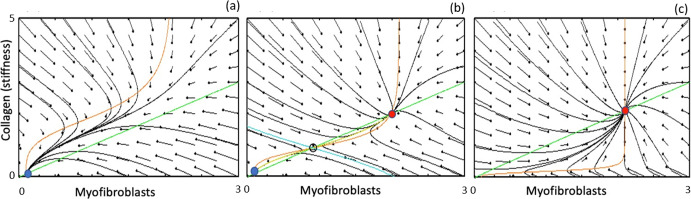
Toy model phase plane analysis: parameters as in Table 1, with three values of the collagen Hill parameter: **a** $C_0=2$, **b** $C_0=1$, **c** $C_0=0.2$ (figure produced by XPP file ToyCC.ode; see “Appendix 7.1”)

The latter (*C* nullcline, green in Fig. 4) is simply a straight line with slope $a/d_C>0$. The former is a sigmoidal curve (*M* nullcline, red in Fig. 4). We show several qualitative outcomes in Fig. 4 from which it is apparent that there can be up to three steady states, the outer two of which are stable.

**Table 1:** Dimensionless parameter values for the toy model

Parameter	Definition	Value
$a_1$	Basal cell recruitment rate	0.1
$a_2$	Collagen-induced cell recruitment rate	2
$d_M$	Cell turnover rate	1
*a*	Rate of collagen production per cell	1
$d_C$	Collagen removal rate	1
*n*	Hill coefficient for *E*(*C*)	4
$C_0$	Collagen Hill parameter	1

There is a range of parameters for which the model is bistable, i.e. for which both low and high steady states coexist. For example, in Fig. 4a–c, we show how the collagen Hill parameter, $C_0$, affects the qualitative behaviour. When $C_0$ is sufficiently high, the system is relatively insensitive to collagen accumulation, that is the collagen feedback *E*(*C*) contribution to amplifying cell recruitment does not suffice to drive the system to an abnormal outcome: only a low steady state value exists, and is the eventual outcome regardless of initial values *M*(0), *C*(0) at time $t=0$. This result implies that women with such relatively insensitive response would never be at risk of developing the CC pathology.

For lower values of $C_0$, the recruitment response switches on at lower collagen density. As shown in Fig. 4b, this case is bistable, so that some initial states (those below the blue “separatrix”, having low *M* and low *C* initially) will resolve toward the normal state, while other initial levels can develop into the abnormal high levels (red dot in *MC* plane). The outcome in such cases is dependent on the situation, and can result in pathology that contributes to capsular contracture. Finally, in Fig. 4c, sensitivity is so fine that even slightly elevated level of *C*, namely $C>C_0=0.2$ results in the pathology.

**Fig. 5:**
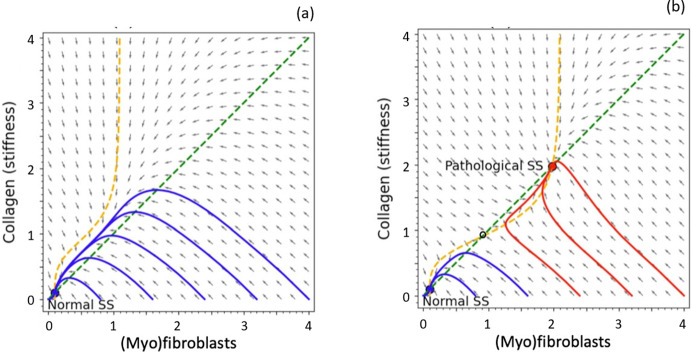
Details of the Toy model phase-plane plots showing scenarios for women with no risk **(a)** and with elevated risk **(b)** of developing capsular contracture. As in Fig. 4a, b, emphasizing the normal steady state outcome (blue) and those that result in the pathological steady state (SS). Parameters as in Table 1, with $C_0=2$ (left), $C_0=1$ (right). The trajectories differ by the initial levels of (Myo)fibroblasts, as shown along the horizontal axes (Color figure online)

**Fig. 6:**
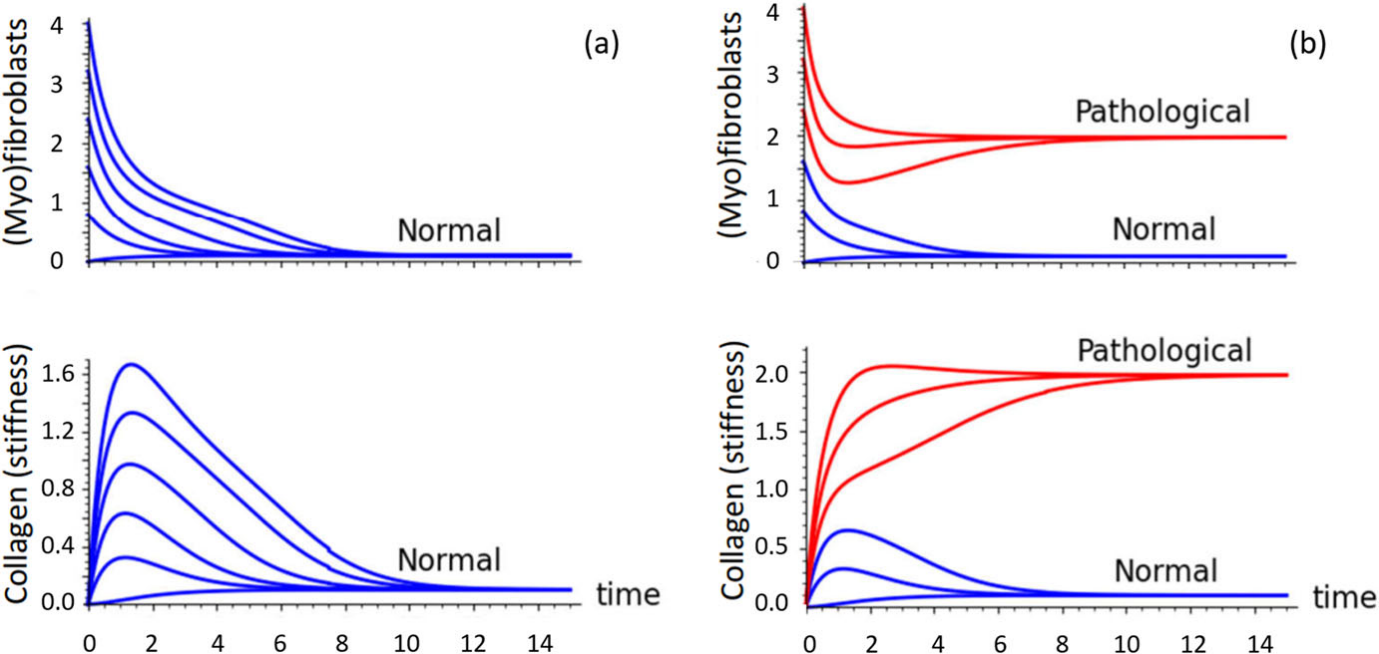
Time behaviour of the cells (top) and collagen (bottom) for the same parameter settings as in Fig. 5a, b. In **(a)** all initial states resolve into a normal low steady state level, whereas in (**b**), some initial states lead to the pathological high steady-state outcome

The specific implications of the model to the capsular contracture are displayed more directly in Figs. 5 and 6. We show two cases in each figure, differing only in the value of $C_0$. In Fig. 5a, only the normal steady state exists, and all initial conditions lead to recovery, as shown by blue curves. In Fig. 5b, the initial state determines the final outcome. Trajectories shown in blue recover, while those shown in red eventually end up in the pathological steady state. Hence, such “susceptible women” are at risk of developing CC. The same distinctions are shown in the time behaviour of Fig. 6, where cell densities and collagen are displayed over time. In Fig. 6a all levels decay to the normal baseline, whereas in Fig. 6b, those that start with some initial conditions evolve towards the high densities of cells and collagen associated with risk of CC.

**Fig. 7:**
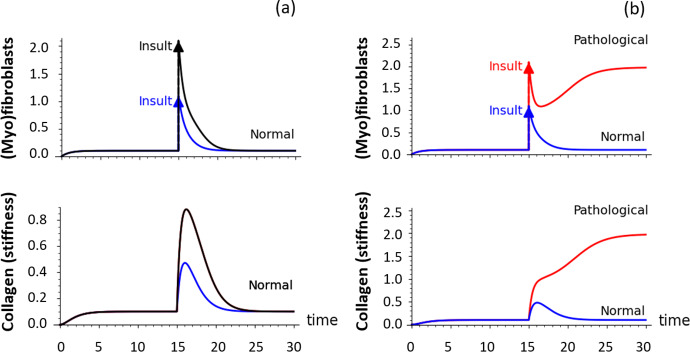
Distinct responses to insult/injury can be obtained from the system. As in Fig. 6, but with initial settings that are consistent with a normal capsule. At $t=15$ a stimulus (e.g. biofilm-induced infection and immune response) is applied. In **(a)** both small and larger insults are resolved back to the healthy normal state, after some transient. In (**b**), the larger stimulus results in the pathological outcome. Model and parameter values as in Fig. 4

Further implications of the model are shown in Fig. 7, where we demonstrate the effect of a perturbation (“insult”) at some stage during their recovery process. This insult may involve one or both breasts. A generalised infection, for example, might induce a bilateral insult, whereas localised trauma (hematoma) due to an accident may only affect one side. In Fig. 7a, there is no risk of CC, even following such an event- full recovery is expected. In Fig. 7b, what appears as a normal recovery can be diverted by a sufficiently large insult, resulting in eventual development of CC.

**Fig. 8:**
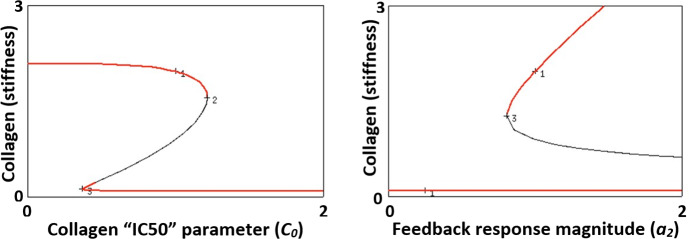
Bifurcation diagrams for the Toy model (3) with respect to the collagen Hill parameter ($C_0$), left, and the response magnitude $a_2$, right. In each case, there is a range of values of the parameter for which low and high stable steady states (red) coexist. There are fold bifurcations associated with the borders of the bistability regimes. Parameter values as in Table 1 (Color figure online)

The simplified model is qualitative, and parsimonious, but demonstrates the underlying idea that feedback to cell recruitment (in this case from collagen, but possibly from other factors) can lead to coexistence of a normal and a pathological state. The following comments provide some context for interpreting the elementary model results.

First, we associate the initial conditions that determine the eventual fate, with some post-surgery scenario, such as excessive inflammation due to surgical factors such as contamination or implant surface properties. Second, details of the feedback function are not important, and similar results are obtained for shallower response function *E*(*C*), e.g, for $n\ge 2$. We chose $n=4$ as it is visually clearer and has a wider range of parameters that correspond to the bistable outcome. Finally, the same conclusions can be obtained by varying another parameter, such as the magnitude of the feedback response, $a_2$, for example. Choosing $C_0=1$, and allowing $a_2$ to take on values $a_2=1.0, 2.0, 4.0$ produces the three basic outcomes shown in Fig. 4.

We show a bifurcation plot with respect to the parameters $C_0$ and $a_2$ in Fig. 8. From these plots we see that low values of $a_2$ or high values of $C_0$ are consistent with no risk of developing CC, since only the normal steady state exists, while high values of $a_2$ or low values of $C_0$ result in either bistability or stability of a high pathological) steady state.

### The Full Model

We now return to the full model, (2), and consider its behavior. In selecting the parameter regime to focus on, we adopt the following strategies.

- We use similar timescales for cells in the full model as for cells in the Toy Model.
- The same response to collagen, *E*(*C*) is considered, but with a sharper switch (Hill coefficient $n=6$) to increase the range of bistability.
- While macrophages enter and leave at some constant rates in this model version, the entry (recruitment) rate $a_\phi$ is a tuning parameter that we associate with the intensity of inflammation.
- We weight the collagen-induced contribution to transdifferentiation to be greater than the macrophage-induced effect.
- We weight the myofibroblast contribution to collagen production to be greater than the fibroblast contribution. This implies that transdifferentiation plays an important role in the collagen accumulation process. The per-cell collagen production rates, *a*, *b* are, respectively $a=2.0$ and 0.1.

**Fig. 9:**
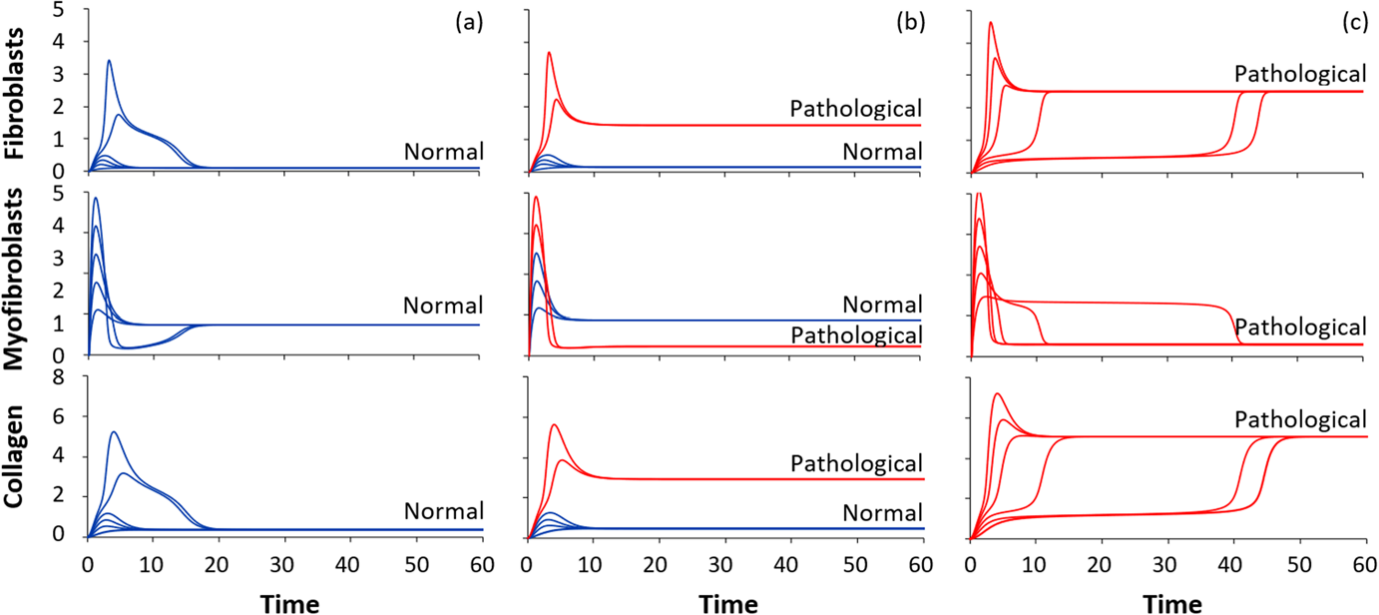
Results of the full model, (2) for various initial inflammatory conditions. The full model was simulated with parameter values as in Table 2, for initial macrophage density of 0,4,8..20), and with increasing inflammatory macrophage recruitment (left to right): **a** $a_\phi =0.5$, **b** $a_\phi =0.8$, **c** $a_\phi =2.0$. In (**a**), there is only a normal steady-state and the collagen density is normal for all immune challenges. In (**b**), a normal and a pathological state coexist, and the initial conditions after surgery determine the eventual outcome. In (**c**), all initial values eventually lead to pathology

**Fig. 10:**
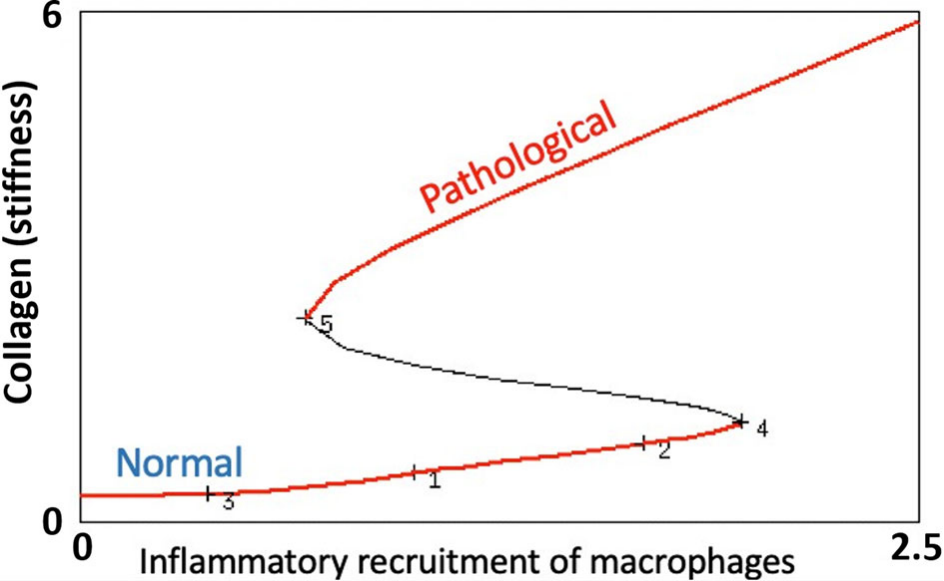
Bifurcation diagram of the full model (2), with respect to the parameter $a_\phi$ that governs the inflammatory recruitment of macrophages to the site of the implant. As in the toy model, the system is bistable over a range of values of $a_\phi$. Low inflammation is consistent with a normal tissue response, while high values lead to the pathology, according to this model. Produced with XPPauto using the XPP file CellsAndCollagenLargerFull.ode in the “Appendix 7.2”

Parameter values are listed in Table 2. Results are shown in Figs. 9 and 10. We focus attention on how the outcome depends on the inflammatory recruitment of macrophages ($a_\phi$). Recall that high $a_\phi$ represents an intense inflammation in response to surgery, recruiting many macrophages to the capsule, and initiating both fibroblast recruitment and transdifferentiation into myofibroblasts.

As in the simpler model, we find distinct possible outcomes. In Fig. 9a with low macrophage recruitment to the site, the system settles into a relatively low, normal level of cells and collagen, for all initial conditions we tested. In Fig. 9b, at higher values of $a_\phi$, the system is bistable. Not only is the macrophage recruitment important, but also the level of cells and/or collagen in the tissue initially determines the outcome: as in the Toy model, the long-term outcomes (steady states) have distinct “basins of attraction”. Finally, in Fig. 9c, at extremely high macrophage recruitment rate, only the pathological state exists. However, as seen in this qualitative model, the system can spend a long time in an intermediate state (with somewhat elevate cell and collagen levels), before reverting to the fully pathological state.

**Table 2:** Base parameter values for the full model

Parameter	Definition	Value
$a_{\phi }$	Macrophages recruitment rate (immune response) [density]/[time]	1
$a_F$	Basal fibroblast recruitment rate [density]/[time]	1
$\beta$	Enhanced fibroblasts recruitment rate per unit macrophage density 1/[time]	1
$a_M$	Basal myofibroblast recruitment rate [density]/[time]	0.1
*a*	Production rate of collagen by myofibroblasts 1/[time]	2
*b*	Production rate of collagen by fibroblasts 1/[time]	0.1
$d_{\phi }$	Macrophage turnover rate 1/[time]	1
$d_F$	Fibroblast turnover rate 1/[time]	1
$d_M$	Myofibroblast turnover rate 1/[time]	1
$d_C$	Collagen turnover rate 1/[time]	1
$a_2$	Magnitude of collagen-enhanced transdifferentiation [density]/[time]	4
$a_3$	Magnitude of macrophage-enhanced transdiftransdifferentiation [density]/[time]	0.1
*n*	Hill coefficient for response to collagen	6
$C_0$	Collagen Hill parameter [density]	2.5
$\phi _0$	Macrophage Hill parameter [density]	1
$m_n$	Hill coefficient for response to macrophages	4

The units of [density] reflect the levels of cells or collagen. These values are used purely for illustrative and qualitative purposes

## Discussion

In this paper, we have presented a series of evidence-based mathematical models of cellular responses to reconstructive breast implants in order to understand the initial causative mechanisms that could lead to capsular contracture. Based on the literature, we developed a broad-scale illustration of the complex cellular responses and signaling that occur following the implant surgery (Fig. 1). We distilled the complex feedback system into the schematic of Fig. 2. We transcribed the schematic into a general model structure given by Eq. (1), tracking macrophage, fibroblast, myofibroblast, and collagen densities ($\phi (t), F(t)$, *M*(*t*) and *C*(*t*).)

After ruling out the simplest linear model as inconsistent with clinical observation, we considered the feedback regulation supported by laboratory and clinical studies including macrophage influence over fibroblast recruitment and transdifferentiation to myofibroblasts. We also introduced regulation of transdifferentiation based on collagen, using collagen density as a correlate of tissue stiffness. We assumed these influences on transdifferentiation were switch-like saturating functions.

To gain qualitative insight into this model, we made some simplifying assumptions to develop a much simplified “Toy Model”. We assumed that in comparison to the time-scale of the capsule formation, the macrophage population rapidly achieved a quasi-steady-state density. We also combined fibroblasts and myofibroblasts into an aggregate population of collagen producing cells with their recruitment being regulated by collagen.

For the present study we are considering qualitative behaviour only. With judicious parameter selection we demonstrate bifurcation with variation of the collagen Hill parameter ($C_0$). At relatively high levels of $C_0$, the patient is insensitive to variability in collagen, and would never be at risk of developing CC. For a “mid range” of $C_0$, a lower level of collagen would amplify cell recruitment, and the response is bistable. For these patients, the ultimate outcome is dependent on the inflammatory conditions either post-op (“initial conditions”, Fig. 6), or in response to some later insult or injury (Fig. 7). Finally, for very small $C_0$, the patient is so sensitive to collagen density that we would expect CC to develop regardless of surgical conditions or exposure to an alternate insult. Similar results are found if $C_0$ is held constant and we vary $a_2$, the collagen-induced cell recruitment rate, with small $a_2$ conferring insensitivity. Both parameters exhibit fold bifurcations (Fig. 8). If we consider the full model, a similar situation arises when we consider variation of the macrophage recruitment rate $a_\phi$: low $a_\phi$ predicts a patient resistant to CC, high $a_\phi$ predicts a patient predisposed to CC, and a moderate $a_\phi$ predicts a patient susceptible to CC, depending on initial or insult-driven elevation in inflammation.

The model has many limitations: many cell types and signalling influences are ignored (some shown in Fig. 1), as are spatial distributions. Timescales are simplified. (See approaches that include many other components in various wound-healing models that are reviewed in Menon and Flegg (2021).) Nevertheless, the models provide a number of insights and sets up a platform for determining (1) candidate features driving CC, (2) conditions that render patients susceptible, and (3) potential preventative measures.

Immune response to surgical contamination or biofilm, subclinical infection, and mechanical factors such as implant architecture have all been implicated in the pathogenesis of CC. The interplay of multiple contributors has complicated elucidating the primary causative factors. Whereas clinical study has tried to elucidate a single prognosticator for CC, here we propose that both the patient’s susceptibility (“inherent parameter values”) and exposure (“initial conditions”) may be required to predict normal versus pathological outcome. A given woman’s genetics and physiology due to extrinsic factors such as co-morbidities, pharmaceuticals, or previous exposure to factors such as radiation, determine her basic “trajectory” (cell responses and interactions). Intrinsic factors such as the breast cancer itself and surgical conditions such as biofilm or contamination affect the initial conditions for the system (initial cell counts, for example.) Under this regime, whereas some patients are resistant to CC initiation (monostable patients), others (bistable patients) may be vulnerable to CC in the presence of elevated cell counts.

This work clarifies and codifies numerous possible clinical interventions. In the bistable “susceptible” population, efforts to keep patients from the basin of attraction to the CC state are paramount, consistent with the clinically observed importance of prophylaxis and sterile technique in avoiding CC. Reducing or eliminating subclinical infection, biofilm, or surgical contamination can be associated with reducing the immune response, represented by the macrophage recruitment rate $a_\phi$. Susceptibility to CC is also modified by increasing the sensitivity of cell recruitment to capsule stiffness. Modulating surface properties such as implant stiffness, texture, and wettability may compensate for fibrotic propensity in some patients by targeting cell adhesion, proliferation, and transdifferentiation on the implant. Given that cell death and collagen clearance also influence susceptibility, other interventions, such as inhibiting myofibroblast apoptosis-resistance or stimulating collagen turnover, could impact the development of CC. Ultimately, interventions which allow a shift in the relevant parameter values (such as pharmaceutical treatment) may shift a patient from monostable CC to bistable, shrink basins of attraction to the CC state for bistable patients, or even shift a patient to the monostable nonsusceptible state.

So far, this first step of modeling the earliest stages of implant surgery failure has focused on the cell recruitment and feedback interactions near an implant. Beyond this first stage, mechanical interactions, tissue deformation, and mechanosensitivity of cells such as myofibroblasts starts to play a dominant role. At that stage, the mechanical properties and deformation of the tissue must be taken into account, aspects that we did not consider in this first step. New feedbacks, including the effect of stress on cell recruitment, and the stress created by cell contractility are then key aspects. To consider these later stages, and the eventual contracture, we are developing, in parallel, a second sequence of models in which such factors are considered in detail. In view of the very distinct flavour and background needed for proper posing of such models, this is done in a separate work.

Although the application of mathematical modeling can prune plausible mechanisms for CC development, further in situ and in vitro studies in the biological system are required to confirm model assumptions. In particular, it would be important to measure the levels of participating cell types and collagen in women who do and who do not develop CC. This can be done at stages where the “expander” temporary implant is replaced by the final implant, work in progress by one of us (KVI).

## Conclusion

Though advances in sterile surgical technique, prophylaxis, and the micro-architecture of breast implants have improved outcomes, capsular contracture remains a painful and disfiguring condition for certain patients after breast-cancer reconstructive surgery. Here we present (to our knowledge) the first mathematical model of early CC etiology. We believe some women are predisposed to fibrosis due to an excessive recruitment of cells and collagen. For these susceptible patients, a local immunogenic insult could cause an abrupt change in the response to their implant and lead to CC. Our model supports that baseline susceptibility may differ across subjects and warrants all patients receive diligent peri-operative prevention for the best outcome.

## Data Availability

Our simulated datasets can be generated by using the code in the “Appendix”, which includes parameter values. Parameters and initial conditions are also stated in the relevant figure caption, and Table 2.

## Appendix

XPP Files used to prepare figures are given below and can also be obtained from an author (LEK) by request.
